# Foot-and-mouth disease (FMD) vaccine market survey at agro-veterinary stores in rural and peri-urban areas of Nigeria

**DOI:** 10.12688/gatesopenres.16349.1

**Published:** 2025-05-20

**Authors:** Adebayo Emmanuel Sopeju, Grace Onoja, Victor Taiwo Abiodun, Andrew R. Peters

**Affiliations:** 1 EMMSAR Veterinary Medical Diagnostic Services, Ibadan, Nigeria; 2 University of Ibadan, Ibadan, Oyo, Nigeria; 3 Arpexas (Scotland) Limited, Moffat, UK

**Keywords:** Foot-and-mouth disease, FMD vaccine, Agro-veterinary stores, Rural and peri-urban areas, Nigeria

## Abstract

Globally, the annual economic impact of foot-and-mouth disease (FMD) is estimated at USD 11 billion in endemic settings, with the impact most profound among smallholder farmers. Farmers and the agro-veterinary stores that supply them are marginalized populations with limited to no access to veterinary care, and paucity of information on the availability and accessibility of vaccines. The objective of this study was to assess the accessibility and distribution channels of FMD vaccine, and the challenges related to the FMD vaccine market in these regions where livestock farming is highest and is an important component of livelihoods.

A cross-sectional study was carried out, where at least one state was selected from five geopolitical zones of Nigeria. The locations were selected because of their high concentrations of livestock farmers. We interviewed 290 agro-veterinary store owners in these locations who directly supply smallholder farmers with animal health products. Data from the interviews were collected through the Kobocollect App ^®^.

Almost all of the agro-veterinary stores in rural and peri-urban areas (96.4%) had direct patronage from livestock farmers. Out of these agro-veterinary stores, relatively few (29%) sold vaccines and among these, 81% did not sell FMD vaccines. More than half (60%) of the stores selling FMD vaccines did not always have the vaccine in stock. Furthermore, maintenance of the cold chain during storage and logistics of the vaccine topped the challenges faced with stocking and sales of livestock vaccines.

It was recommended that to ensure FMD control by the livestock farmers in rural and peri-urban areas through the use of FMD vaccines sold by agro-veterinary stores located in their communities, there is a need to adopt some, if not all, of the suggestions provided by the agro-veterinary store owners. This will ultimately improve animal productivity, and farmer livelihoods, and contribute to national food security.

## Introduction

Foot-and-mouth disease (FMD) is a viral infection that affects cloven-hoofed livestock and wildlife. Important livestock hosts of FMD virus are cattle, pigs, goats, sheep, yaks, and water buffalo ( Jamal and Belsham, 2013). The virus, foot-and-mouth disease virus (FMDV), belongs to the genus *Aphthovirus* and the family *Picornaviridae.* It is a non-enveloped, single-stranded, positive sense RNA virus of approximately 8.5kb in size. There are seven serotypes of FMDV, namely O, A, C, SAT 1, SAT 2, SAT 3, and Asia 1 ( Jamal and Belsham, 2013). In Nigeria, where the disease is endemic with seasonal outbreaks, the circulating serotypes are O, A, SAT 1 and SAT 2, with serotype O the most prevalent ( FAO, 2021). The disease is characterized by high morbidity and low mortality (except in calves) with devastating economic losses due to the debilitating nature of the disease. The major lesions are vesicles in the oral cavity and hoof resulting in erosion and ulceration in these areas. These lesions lead to hypersalivation, unwillingness to eat, and reluctance to stand or walk in the affected animal causing decreased production.

Globally, the annual economic impact of FMD is estimated at USD 11 billion in endemic settings. The economic impact is most profound among smallholder farmers as the disease threatens their livestock productivity, income, livelihoods and food security ( Alhaji *et al., *2020).

To control the disease, inactivated vaccines made from Nigerian-endemic strains have been shown to be effective for protection in cattle against FMD ( Mowat *et al., *1975; Lazarus *et al.*, 2012). However, the vaccine is neither readily available nor popularly known among livestock farmers, causing a huge loss especially among smallholder farmers who often lack awareness of the vaccine and its benefits ( FAO, 2012).

The economic impact of FMD can be viewed as direct or indirect, with both affecting the farmers’ returns on investment. Reduced or loss of milk production, mortality, especially in calves, loss of weight and draft power, and reduced livestock market value are the direct impacts ( Senturk and Yalcin, 2005; Barasa *et al.*, 2008; FAO, 2012; Young *et al.*, 2013). Indirect impact is seen in expenditure on disease control through vaccination, diagnostics and surveillance, treatment of secondary bacterial infections, and movement control ( FAO, 2012). Although returns on investment in cases of FMD control (using FMD vaccine) have been seen to be positive, with a benefit-cost ratio of 33.6 as reported by Alhaji *et al.*, (2020), the intentional use of the vaccine is very unpopular among farmers in Nigeria, especially the smallholder farmers who are known to keep more than 10 animals (cattle and small ruminants) ( Peters *et al., *2012). The major resultant effect for livestock owners is the embargo on the importation or exportation of meat from animals in FMD-affected areas ( Ezeokoli *et al., *1988).

Based on the findings of Alhaji *et al.* (2020) and Peters *et al.* (2012) that reported that FMD vaccination is an unpopular practice among smallholder farmers, this study aims to understand the market dynamics of FMD vaccine at the agro-veterinary stores in rural and peri-urban areas, gather information on the challenges faced by agro-veterinary stores and how they can be supported to ultimately ensure that smallholder farmers routinely use FMD vaccine to control the disease in Nigeria. This study is relevant and crucial, for should there be any effective FMD control in Nigeria, these areas with highest concentrations of livestock, which are often neglected, should be the main focal points.

## Methods

**Figure 1.**
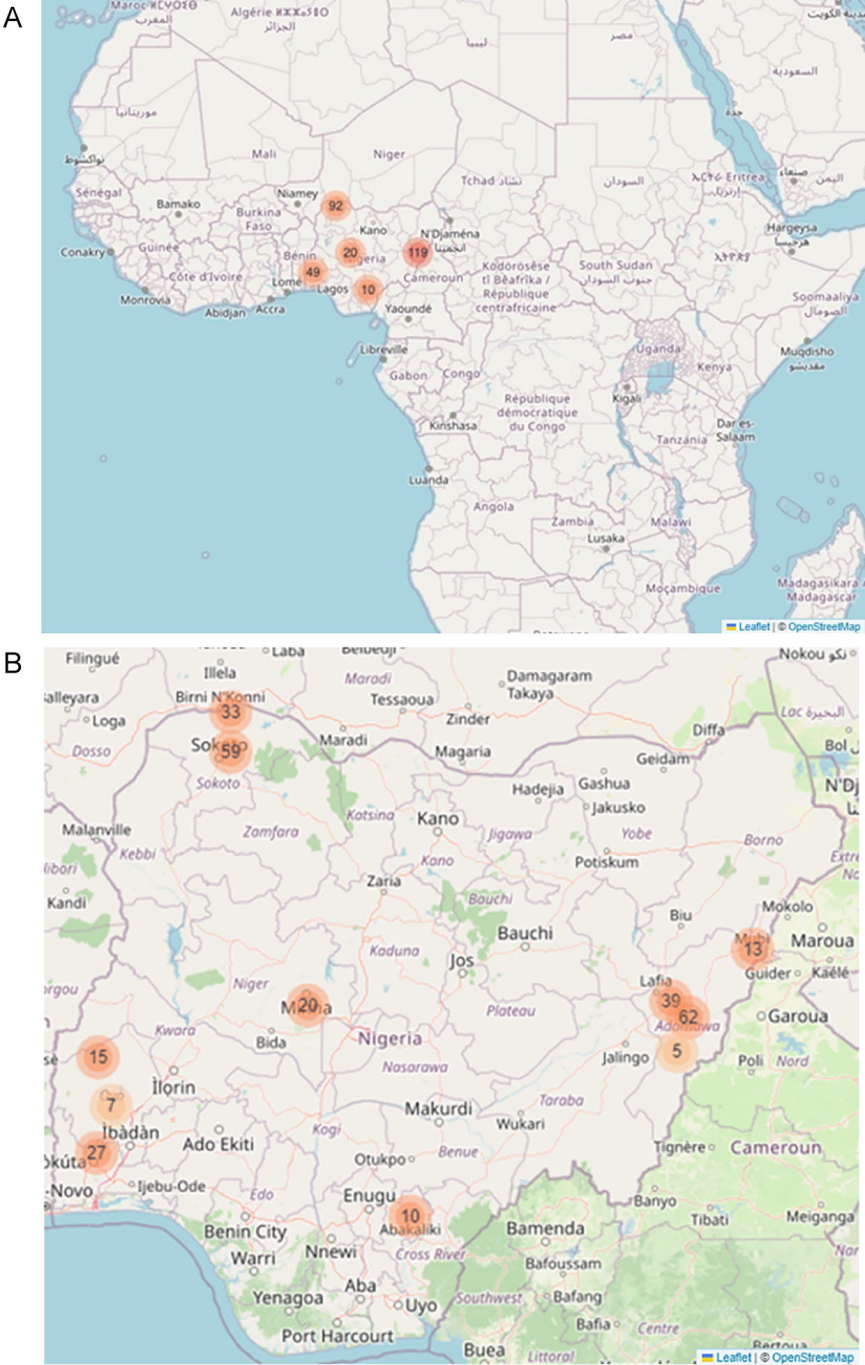
Location of study areas; A) The map shows the position of Nigeria within Africa, and the five geopolitical zones selected; B) The map shows the towns where the agro-veterinary stores are located. OpenStreetMap was used to create the maps; OpenStreetMap is open data, licensed under the Open Data Commons Open Database License (ODbL) by the OpenStreetMap Foundation (OSMF), https://www.openstreetmap.org/copyright . The orange circles indicate the number of agro-veterinary stores per location.

**Study areas:** We selected a minimum of one state from five out of the six geopolitical zones of Nigeria, between November to December 2023. Most of the states selected are located far away from urban areas within each state. The selected states from each of the five geopolitical zones are found in Table 1 and Figure 1.

**Table 1.** Geopolitical zones and states selected for the project.

S/N	Geopolitical zones	States
**1.**	South-East	Ebonyi
**2.**	South-West	Ogun and Oyo
**3.**	North-Central	Niger
**4.**	North-West	Sokoto
**5.**	North-East	Adamawa

**Study site:** We purposively selected locations relatively distant from the state capital and popular cities within the state. Interviews were conducted at agro-veterinary stores located in rural and peri-urban areas that supply smallholder farmers with animal health products.

**Data collection:** The questionnaire (Extended Data), composed of both closed and open-ended questions, was uploaded on Kobocollect App ^®^. It was read out in-person to participants in the most preferred language (English or local language) by licensed animal health service providers (veterinarians or animal health technologists). Key informant one-to-one interviews were recorded on tape to obtain information on important factors that may not have been captured by the questionnaires, and to make the discussion with the stakeholders as interactive as possible ( Elelu, 2017; Chemuliti *et al.*, 2021). Questionnaires and interviews were conducted in agro-veterinary stores and the location coordinates of each participating agro-veterinary store were recorded by global positioning system (GPS) but all participant details were anonymised.

**Sample size:** In total, we interviewed 290 agro-veterinary store owners from 35 local government areas of six states of Nigeria. We also conducted 65 oral interviews with the key informants among community leaders, livestock farmers, and agro-veterinary store owners.

**Data analysis:** The data were entered into Microsoft Excel 2010 (Microsoft Corporation) software for data cleanup, using a licensed version of Microsoft Office Excel, and later exported to IBM SPSS ^®^ statistics version 25 software (IBM Corp.) for statistical analysis. Chi-square or Fisher’s exact test (as the number counts in some cells were less than five) was used to test the significance of associations between demographic features and business operations using GraphPad Prism 8.4.2. The level of significance was set at a value of p < 0.05.

**Ethical consideration:** There is no legal requirement in Nigeria for review by an Ethics Committee of such a study. However, since it was decided to aim to publish in Gates Open Research, it was felt that a retrospective review would be appropriate (rather than none at all). Ethical approval for the project, questionnaire, consent forms and other participant information materials was obtained from the University of Ibadan (UI) UCH Ethics Committee of the Institute for Advanced Medical Research and Training (IAMRAT), College of Medicine, University of Ibadan (registration number: NHREC/05/01/2008a; UI/UCH Ethics Committee assigned number: UI/EC/24/0256), dated 08/04/2024 to 07/04/2025.

### Consent to participate

Informed written consent was obtained from all participants before the questionnaire was administered.

## Results

Of the 290 participants who gave their consent to participate in the study, Adamawa State had the highest number of participants with 49%, while the least was in Ebonyi State with 3% of all participants ( Table 2).

**Table 2.** Local government areas and states selected for the project.

S/N	State	LGA	Number	Percentage
**1.**	Adamawa	Demsa	18	15%
		Girei	13	11%
		Guyuk	3	3%
		Jada	4	3%
		Lamurde	2	2%
		Mayo Belwa	14	12%
		Mubi	13	11%
		Numan	22	18%
		Shelleng	7	6%
		Yola North	3	3%
		Yola South	20	17%
		**Total**	**119**	* **41%**
**2.**	Sokoto	Batsari	4	3%
		Bodinga	20	17%
		Gada	11	9%
		Goronyo	10	8%
		Illela	21	18%
		Kware	2	2%
		Raba	2	2%
		Sabon Birni	2	2%
		Sokoto North	2	2%
		Sokoto South	1	1%
		Wurno	17	14%
		**Total**	**92**	* **32%**
**3.**	Niger	Bosso	6	30%
		Chanchanga	3	15%
		Paikoro	11	55%
		**Total**	**20**	* **7%**
**4.**	Oyo	Ibarapa Central	3	10%
		Ibarapa East	5	17%
		Iseyin	7	23%
		Saki-West	15	50%
		**Total**	**30**	* **10%**
**5.**	Ogun	Abeokuta North	3	15%
		Abeokuta South	7	35%
		Odeda	9	45%
		**Total**	**19**	* **7%**
**6.**	Ebonyi	Abakaliki	6	60%
		Ebonyi	3	30%
		Ohaukwu	1	10%
		**Total**	**10**	* **3%**
		**Grand total**	**290**	**100%**

* This figure represents the percentage of the total participants in each state as compared with the total participants from all states.

### Gender distribution

Among the 290 agro-veterinary store owners interviewed, 274 (94.5%) were male, while 16 (5.5%) were female ( Table 3). Although the proportion of females managing agro-veterinary stores in the southern region (62.5%) was significantly more than in the northern region (37.5%), the reverse was observed among the males (South – 17.9%; North – 82.1%) ( Table 4). Of all the study states, Ebonyi and Oyo had the highest percentage of female agro-veterinary store owners (each having 20.0%) while Sokoto State had no female agro-veterinary store owners.

**Table 3.** Gender of agro-veterinary store owners.

State	Adamawa (%)	Niger (%)	Sokoto (%)	Ebonyi (%)	Ogun (%)	Oyo (%)	Total (%)
**Male**	66 (93.4)	18 (90.0)	141 (100.0)	8 (80.0)	17 (89.5)	24 (80.0)	**274 (94.5)**
**Female**	4 (5.6)	2 (10.0)	0 (0)	2 (20.0)	2 (10.5)	6 (20.0)	**16 (5.5)**
**Total**	**70 (100.0)**	**20 (100.0)**	**141 (100.0)**	**10 (100.0)**	**19 (100.0)**	**30 (100.0)**	**290 (100.0)**

**Table 4.** Gender involvement in agro-veterinary business based on Region in Nigeria.

Region	North	South	Total
**Male**	225 (82.1%)	49 (17.9%)	**274 (94.5%)**
**Female**	6 (37.5%)	10 (62.5%)	**16 (5.5%)**
**Total**	231 (79.7%)	59 (20.3%)	**290 (100%)**

P<0.05 (p=0.0002).

### Educational qualification

**Figure 2.**
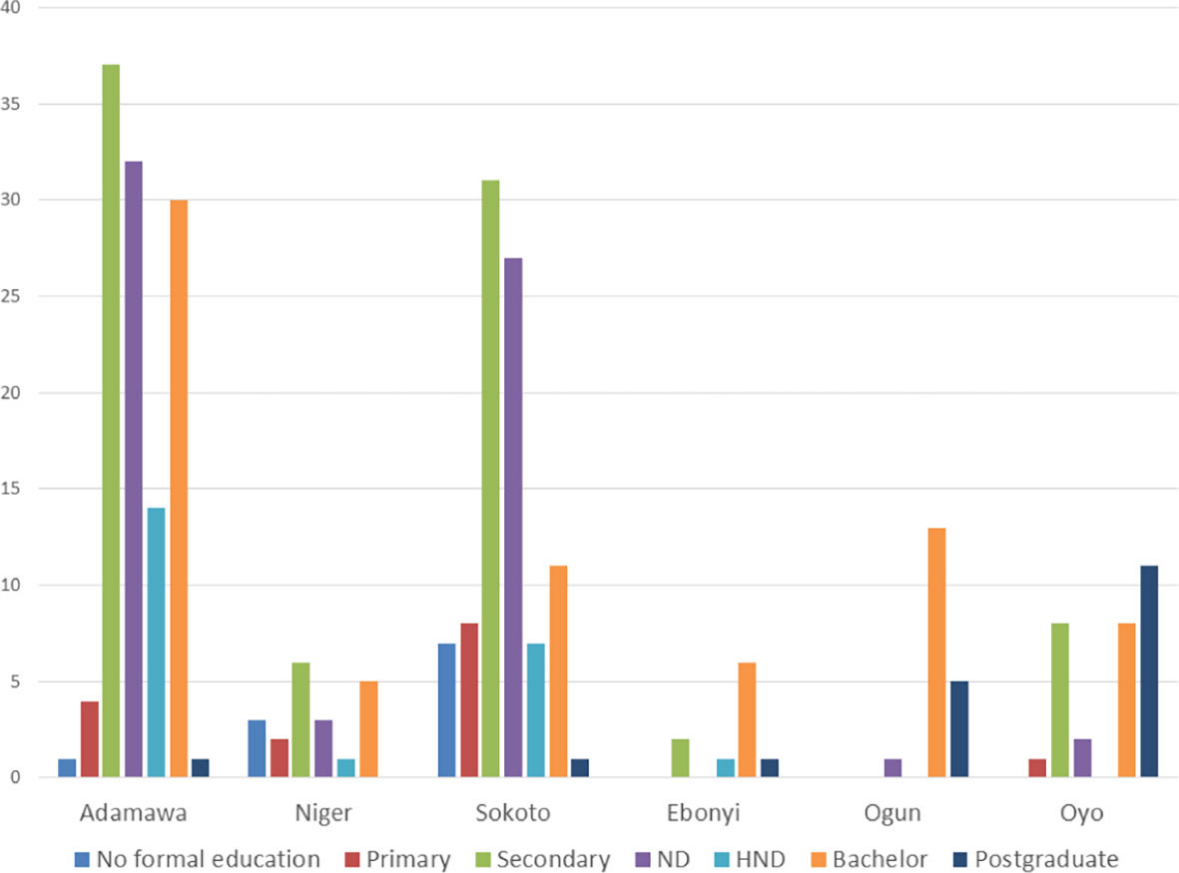
Educational status of agro-veterinary store owners per state, showing the highest level of education of the store owners.

**Figure 3.**
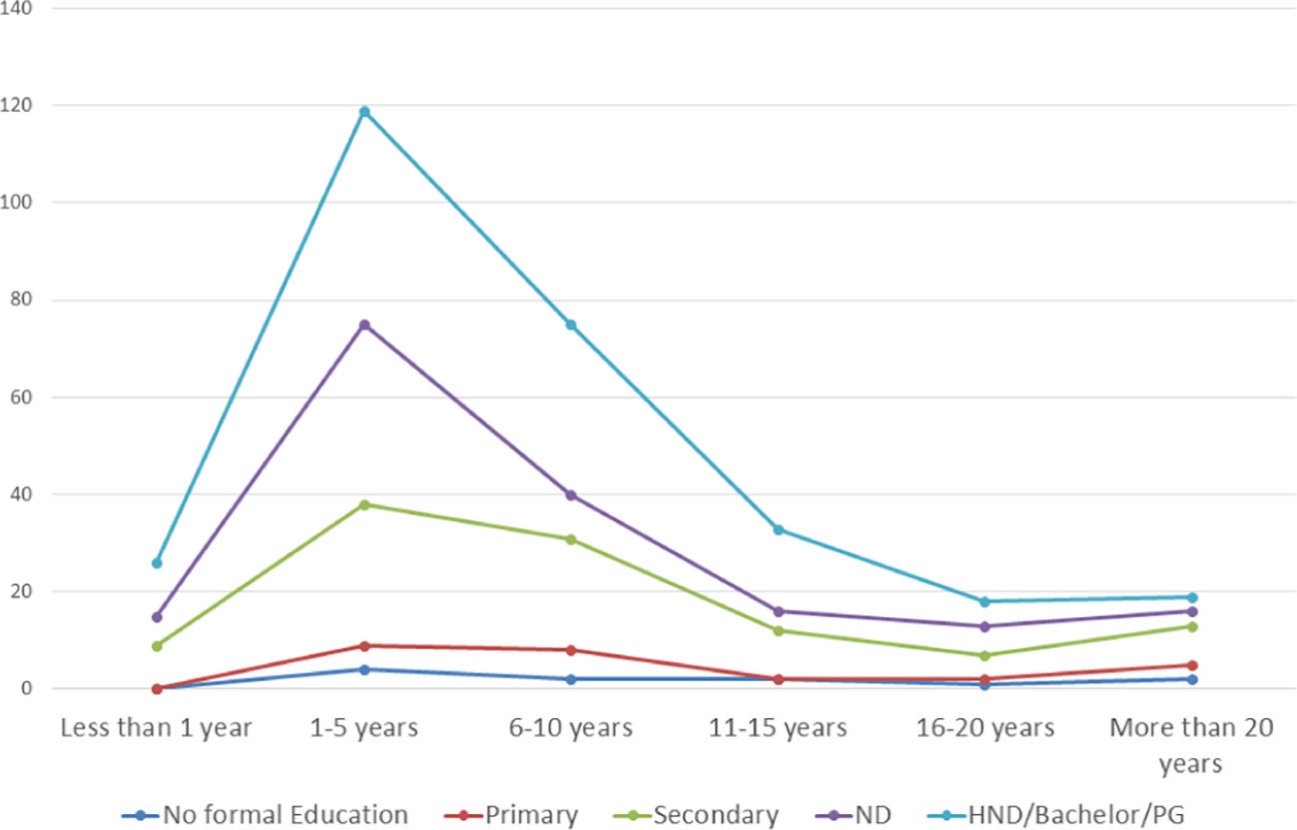
Highest educational qualification of agro-veterinary stores over time.

The educational qualifications of agro-veterinary store owners interviewed ranged from no formal education to postgraduate degree, with a moderate number of the participants (approximately 40%) having at least a graduate educational qualification (HND, bachelor or postgraduate). As seen in Figure 2, most participants from all of the states in the northern region have secondary school certificates as the highest educational qualification, while in the southern region, most of the participants have degree certificates as their highest qualification. The highest number of participants with postgraduate certificates was found in Oyo State. Furthermore, we realized in this study that more educated individuals (with at least a graduate certificate as the highest educational qualification) are joining the agro-veterinary business in the peri-urban and rural areas as seen in Figure 3.

### Years of operation

**Figure 4.**
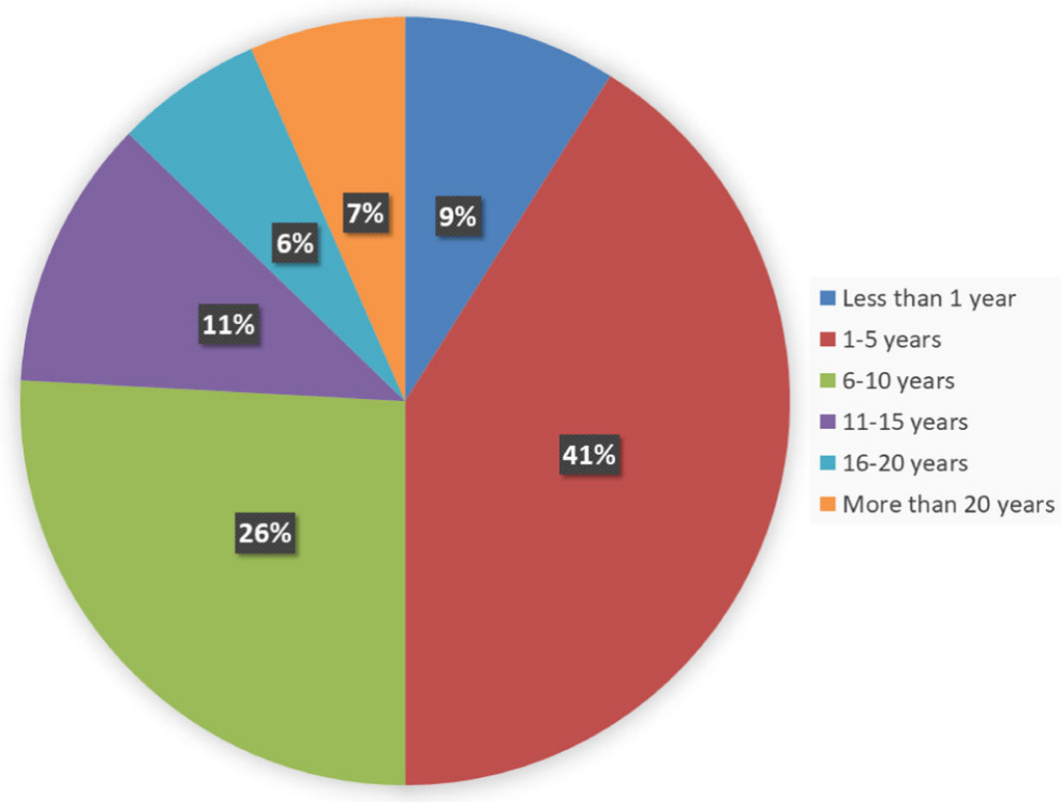
Years of agro-veterinary business operations.

The agro-veterinary store owners interviewed had varied years of agro-veterinary business operations, as seen in Figure 4. The largest proportion of store owners (41%) have been in the agro-veterinary business operation in the last 1-5 years.

### Vaccine marketing by the agro-veterinary stores

**Figure 5.**
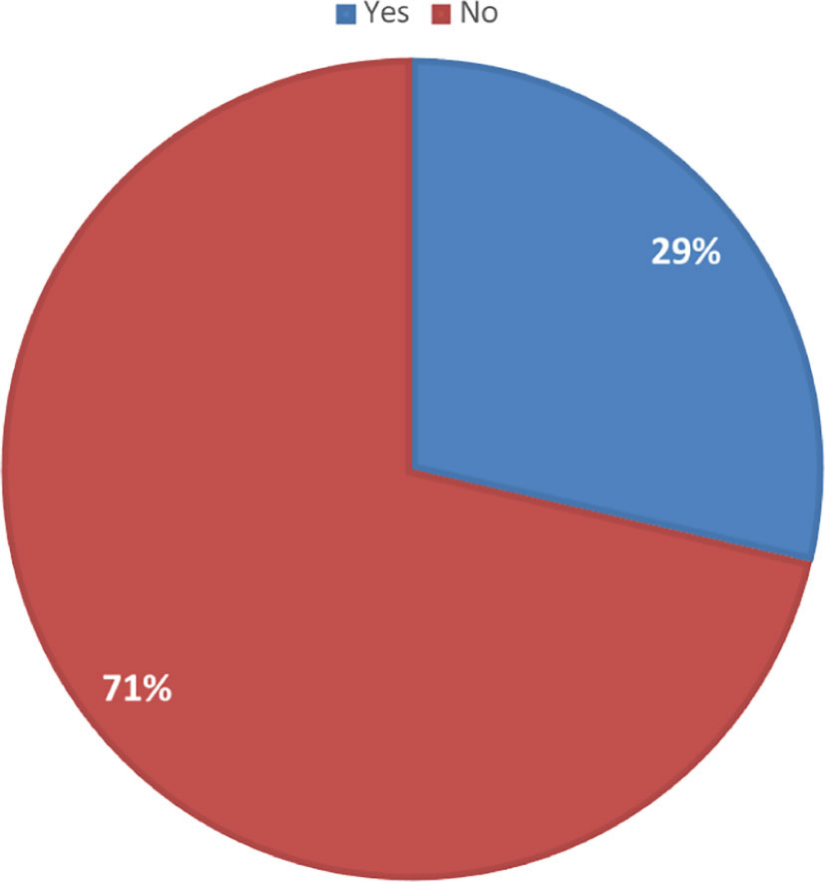
Percentage of agro-veterinary stores selling vaccines in the rural and peri-urban areas.

**Figure 6.**
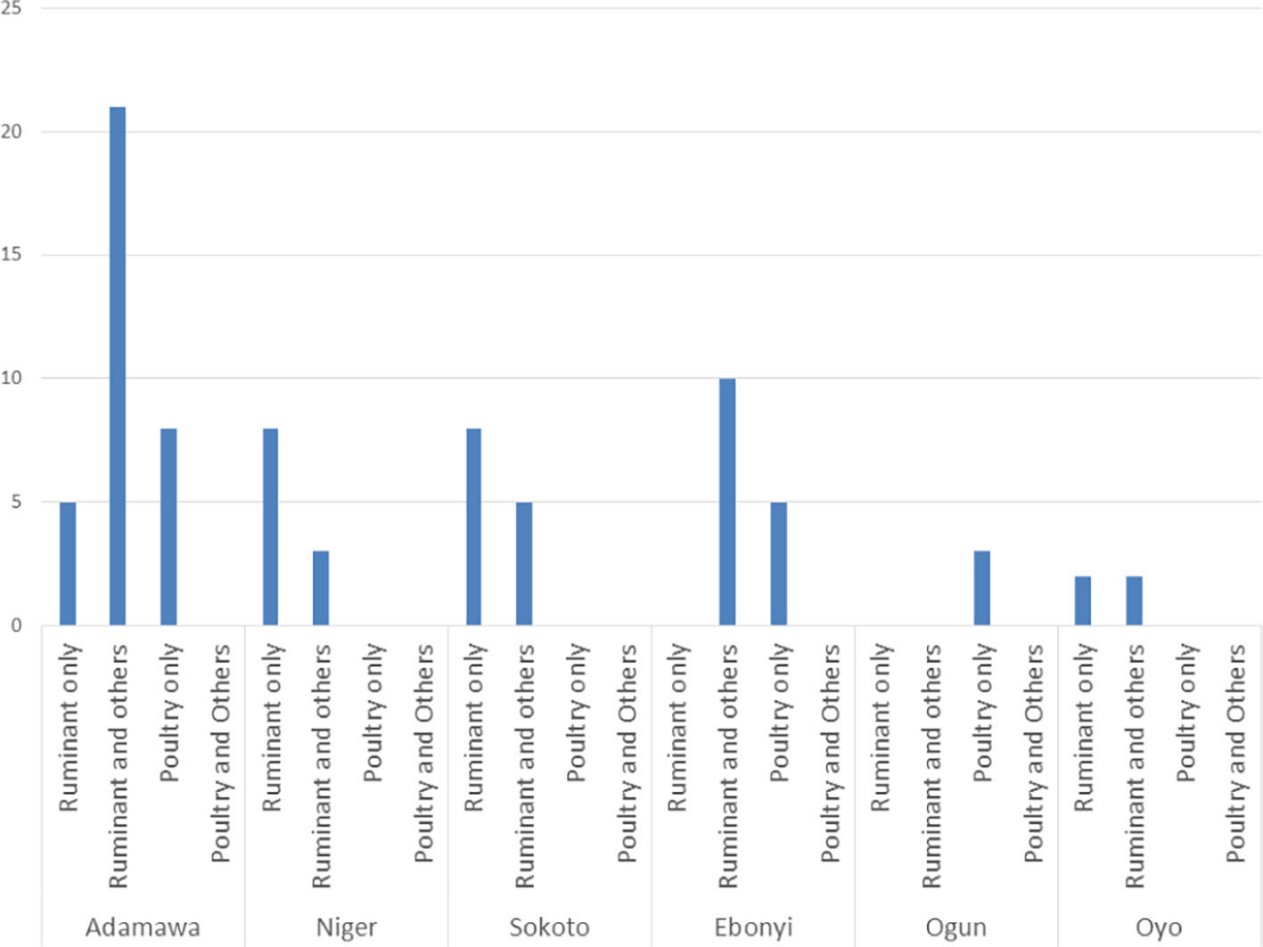
Categories of vaccine sold by agro-veterinary stores per state.

The study identified that out of the agro-veterinary stores in peri-urban and rural areas, only a few (29%) sell vaccines, including ruminant vaccines, as seen in Figure 5. From Table 5, it is clear that the percentage of agro-veterinary stores selling vaccines in southern states (Ebonyi, Ogun, and Oyo) is significantly more than those selling in the northern states (Adamawa, Niger, and Sokoto). Similarly, there were more stores selling ruminant vaccines alone or with other vaccine types in the northern states than the southern states ( Figure 6).

**Table 5.** Geographical distribution of agro-veterinary stores selling vaccines per state and region.

State	Yes	No	Total
**Adamawa**	31	88	119
**Sokoto**	8	84	92
**Niger**	8	12	20
**Ogun**	11	8	19
**Oyo**	16	14	30
**Ebonyi**	9	1	10
	83 (29%)	207 (71%)	290 (100%)
**P<0.05; (p=0.0001)**			
**Region**	Yes	No	Total
**North**	47	184	231
**South**	36	23	59
	83 (29%)	207 (71%)	290 (100%)

P<0.05; (p<0.0001).

### Marketing of FMD vaccine by the agro-veterinary stores

Of all the agro-veterinary stores that were selling animal vaccines, more than 81% were not selling FMD vaccines i.e., only 19% selling FMD vaccines. Of all the stores selling FMD vaccines, nine stores only sometimes had the vaccine, only three had the vaccine in stock frequently, and only one store always had the vaccine. Almost all of the agro-veterinary stores in rural and peri-urban areas (96.4%) selling vaccines had direct patronage from farmers ( Table 6).

**Table 6.** Sales of FMD vaccine by agro-veterinary stores.

Agro-veterinary store selling vaccines	Frequency (n=209)	Percentage (%)
**Yes**	83	28.6
**No**	207	71.4
**Agro-veterinary selling FMD**	**Frequency (n=83)**	
**Yes**	15	18.1
**No**	68	81.9
**Availability of FMD Vaccine**	**Frequency (n=15)**	
**Always**	1	6.7
**Frequently**	3	20.0
**Sometimes**	9	60.0
**Never**	2	13.3
**Customers**	**Frequency (n=83)**	Percentage
**Farmers**	80	96.4
**Veterinarians**	41	49.4
**Animal health officer**	39	47.0
**Government**	11	13.3
**Others**	4	4.8

### Sources of vaccine

To understand the source of vaccines sold in stores, we asked about sources of vaccine e.g. manufacturer or veterinary clinic ( Table 7). More than 84% of the agro-veterinary stores sourced their vaccines through distributors or marketers, followed by government programs, although this was significantly more popular in the northern region than the southern region. The smallest proportion sourced vaccines from veterinary clinics (15.7%) ( Table 7). Additionally, 73.5% of the agro-veterinary stores got their vaccines transported by commercial bus or car traveling from urban areas to rural or peri-urban areas, while some organizations got the vaccine to these agro-veterinary stores through their sales representatives or field marketers, as shown in Table 8.

**Table 7.** Sources of vaccine supply to agro-veterinary stores in rural and peri-urban areas.

		Sources of vaccines		
	Manufacturer	Veterinary Clinic	Through distributors	Through Govt program
**Adamawa**	10	8	27	14
**Ebonyi**	1	0	8	1
**Ogun**	3	1	11	1
**Oyo**	4	2	14	2
**Sokoto**	0	2	6	1
**Niger**	0	0	4	6
**Total**	18	13	70	25
**Percentage**	21.7%	15.7%	84.3%	30.1%
**Region**	Manufacturer	Veterinary Clinic	Through distributors	Through Govt program
**North**	10	10	37	21
**South**	8	3	33	4

P<0.05 (p=0.0273).

**Table 8.** Transportation of vaccine to agro-veterinary stores in rural and peri-urban areas.

	Means of transportation of vaccine		
	Commercial car/bus (n=83)	Motorcycle (n-83)	Though Sales Rep (n-83)
**Adamawa**	25	1	22
**Ebonyi**	9	0	0
**Ogun**	2	0	10
**Oyo**	12	4	2
**Sokoto**	8	1	0
**Niger**	5	7	1
**Total**	61	13	35
**Percentage**	73.5%	15.7%	42.2%

### Vaccine cold chain maintenance in transportation and storage

The study showed that there were no agro-veterinary stores receiving their vaccines from suppliers under an optimum cold environment at all times ( Table 9). However, most of them (54.2%) frequently had vaccines delivered to them under a standard cold environment. Fewer than half (45.8%) of agro-veterinary outlets always stored their vaccine in cold environments.

**Table 9.** Vaccine cold chain maintenance in rural and peri-urban areas.

	Vaccines delivered in Cold Chain (n=83)	Are the vaccines stored in cold environment (n=83)
**Always**	0 (0%)	38 (45.8%)
**Frequently**	45 (54.2%)	31 (37.3%)
**Sometimes**	25 (30.1%)	11 (13.3%)
**Never**	2 (2.4%)	2 (2.4%)
**When the vaccine requires it**	0 (0%)	1 (1.2%)

### Factors that affect agro-veterinary stores no longer selling FMD vaccine

Different reasons contributed to the stoppage of sales of FMD vaccines as seen in Table 10; the vaccine was expensive, there was a problem maintaining the cold chain, and farmers were not requesting the vaccines which made some of the vaccines expire, to mention a few.

**Table 10.** Factors that affected agro-veterinary stores that no longer sell FMD vaccine.

Agro-veterinary store that once sold FMD but no longer sell FMD vaccines				
State	(n=9)	Duration (n)	Education (n)	Reasons (n)
**Adamawa**	2	1-5 years (2)	Primary (1)	Farmers are not requesting for it (2)
			ND (1)	It is too expensive (2)
				My source of getting vaccine no longer sells FMD vaccine (1)
				It is no longer available (1)
				I bought some that expired before I could sell them (1)
**Niger**	4	6-10 years (1)	No formal education (1)	I don't have a refrigerator to maintain the cold chain (1)
		11-15 years (1)	Secondary (2)	The FMD vaccines are not effective (1)
		More than 20 years (2)	HND (1)	Farmers are not requesting for it (1)
				It is no longer available (2)
**Oyo**	1	1-5 years	Postgraduate	It is no longer available (1)
**Ogun**	1	11-15 years	Bachelor	Farmers are not requesting for it (1)
				It is too expensive (1)
				It is no longer available (1)
**Sokoto**	1	16-20 years	ND	Farmers are not requesting for it (1)

**Figure 7.**
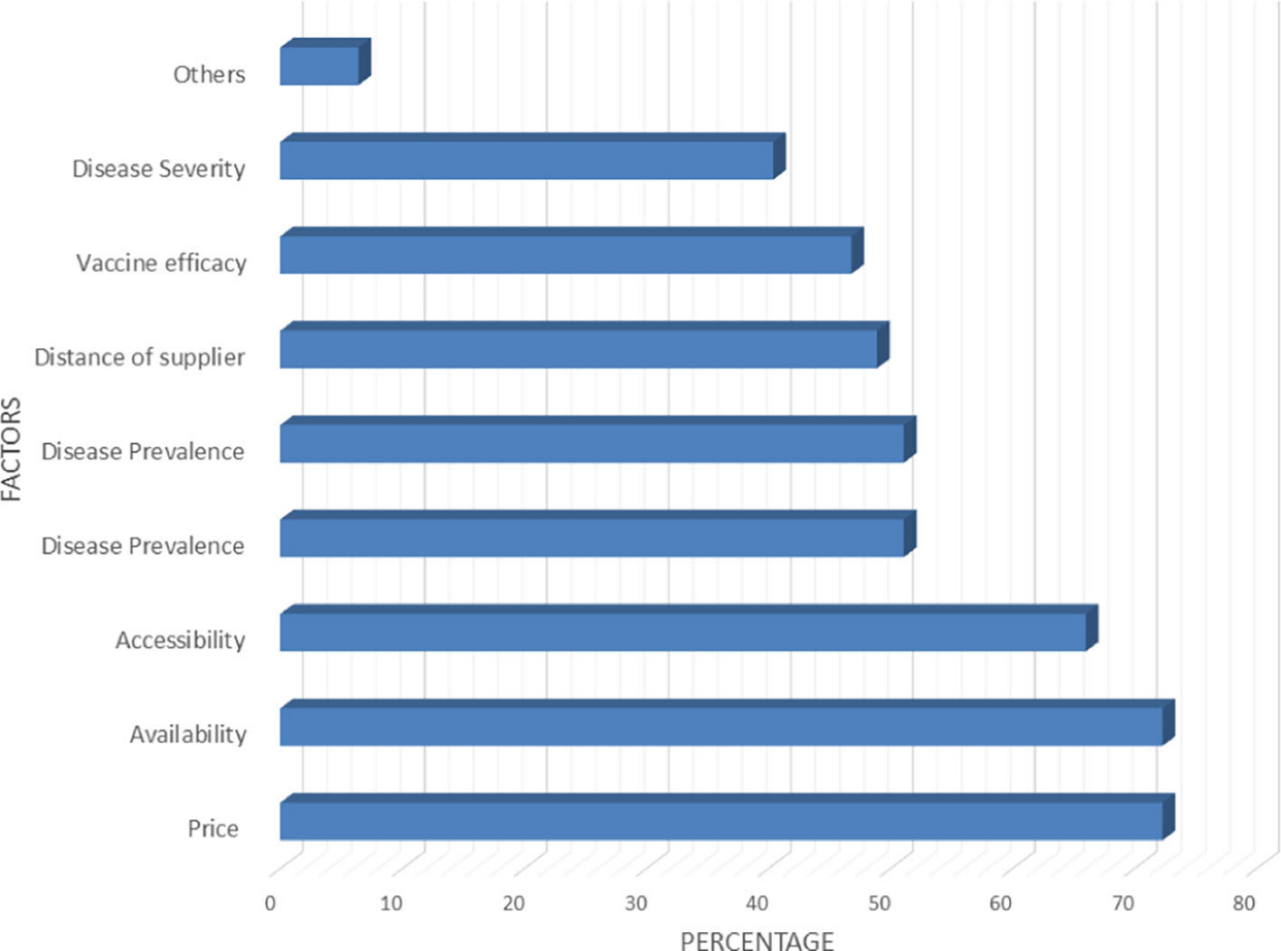
Factors that dictate the type of vaccine purchased by agro-veterinary stores in rural and peri-urban areas.

Among the factors that determine the type of vaccines that are purchased by agro-veterinary store owners in rural and peri-urban areas, the price of the vaccine and its availability in the market were the strongest ones, followed by accessibility of vaccines ( Figure 7). More than 44% of the agro-veterinary stores located in rural and peri-urban areas had to travel more than two hours before they could procure their vaccines of choice ( Table 11). A similar finding was reported by Chemuliti *et al.* (2021), reporting 33% of the agro-veterinary stores had challenges with long-distance sources of their vaccine.

**Table 11.** Travel duration of agro-veterinary stores to vaccine sources.

Time spent when traveling to get the vaccine		
Time	N =83	Percentage (%)
**Less than 30 minutes**	3	3.6
**30 min-1 hr**	7	8.4
**1-2 hrs**	20	24.1
**Over 2 hrs**	37	44.6
**Not sure**	16	19.3

Of all the participants selling vaccines, 77 (92.8%) reported their constraints of sales of FMD vaccines by agro-veterinary stores in the rural and peri-urban. Maintenance of the cold chain during storage and logistics of the vaccine to livestock farmers topped the challenges with 32.5% of respondents. The respondents further explained that no or low power supply, no refrigerator, the high cost of running a generator and the absence of materials to maintain the cold chain during the logistics of the vaccine to the farmer discouraged not only the agro-veterinary stores but also the livestock farmers as these factors affect vaccine efficacy. Other challenges mentioned were lack of knowledge of the impact of the disease and the benefits of the vaccine among livestock farmers (27.3%), high cost of vaccine (22.1%), frequent unavailability of the vaccine when ordered (9.1%), distance of the farmers to agro-veterinary stores (7.8%), lack of vehicle for logistics of the vaccine to the farmers (6.9%) and poor knowledge of the administration of FMD vaccine (3.9%), among other factors ( Table 12).

**Table 12.** Challenges to FMD vaccine sales by agro-veterinary stores in rural and peri-urban regions.

Participant that responded			
	Frequency (n=83)	Percentage (%)	
**Yes**	77	92.8	
**No**	6	7.2	
**Challenges**	Frequency (n=77)	Percentage (%)	Challenge types
**Cold chain**	25	32.5	- No or low power supply - No refrigerator - High cost of running on generator - No facility to maintain cold chain during supply
**Lack of knowledge**	21	27.3	- Impacts of FMD - Value of FMD vaccine - Effective brand of vaccine
**Price of the vaccine**	17	22.1	- FMD vaccine is expensive - High forex affecting the cost of vaccine - Frequent hike in FMD vaccine price
**Unavailability of vaccine**	7	9.1	- Not available at all - Delayed supply if available
**Distance of the farmers to agro-veterinary stores**	6	7.8	- Distance of farms/farmers to agro-veterinary stores - Distance of farm owners to the animals for the decision to buy the vaccine
**Transportation**	5	6.5	- No vehicle to transport the vaccine - Poor road network
**Vaccine administration**	3	3.9	- Poor knowledge of the route of administration - Poor knowledge of the vaccine regime - Poor knowledge of the correct dosage
**Vaccine efficacy**	2	2.6	- Prevalence of cheap, fake FMD vaccine - Poor vaccine efficacy
**Alternative therapy**	2	2.6	- Trust in communal FMD treatment approach - General preference of treatment of FMD to vaccination
**Seasonal market/demand**	2	2.6	- FMD is a seasonally prevalent disease - Demand for vaccines is seasonal - FMD vaccine is perceived to have a short expiry date
**Sourcing the vaccine**	2	2.6	- Difficulty in sourcing for the vaccine - Unavailability of lower doses from the source
**Security**	1	1.3	- Affecting reaching some areas for the supply of the vaccine - Affecting decision-making of animal care generally
**Lack of capital**	1	1.3	- Buying and storing FMD vaccine is capital-intensive - No funds to get FMD vaccine
**Distrust in government**	1	1.3	

## Discussion

The study aimed to understand the market dynamics of FMD vaccine at the agro-veterinary stores in rural and peri-urban areas, and gather information on the challenges faced by agro-veterinary stores. The structured questionnaire captured data on several areas, including socioeconomics such as gender and education of agro-veterinary owners. It was observed that the proportion of female agro-veterinary store owners (5.5%) was significantly lower than the male store owners (94.5%). This is similar to the finding of Elelu (2017) that reported that among agro-veterinary store owners, 96.3% were male (3.7% female). The slight difference may be due to the state selected in their study (Kwara state) which falls under the north-central part of Nigeria. Furthermore, there were more women owning agro-veterinary stores in the southern region than in the northern region. This may be due to many factors, most notable being religion, cultural background and level of education ( Enfield, 2019). The study found that the highest number of participants with postgraduate certificates was found in Oyo State. This is in contrast with the study that reported that most of the owners of agro-veterinary stores have either primary or no formal education ( Elelu, 2017). This variance may be due to the time the study was conducted or the states of the country where the study was carried out. The observation that more educated individuals (with at least a graduate certificate as the highest educational qualification) are joining the agro-veterinary business in the peri-urban and rural areas is a new development, which coincides with the introduction of training of veterinary paraprofessionals in Nigeria; if well appropriated, this could provide a solution to the common practice of veterinary healthcare delivery by untrained, unprofessional owners of agro-veterinary stores in the rural and peri-urban areas ( Grasswitz *et al.*, 2004; Faramade *et al.*, 2016).

The largest proportion of store owners (41%) were found to have been in the agro-veterinary business operation in the last 1-5 years; this suggests that more people are taking an interest in and starting an agro-veterinary business. We found that there were more stores selling ruminant vaccines alone or with other vaccine types in the northern states than the southern states. Previous studies have shown that agro-veterinary stores would rather procure and sell veterinary drugs to livestock farmers ( Grasswitz *et al., *2004; Elelu, 2017) instead of vaccines as there are several limiting factors to the sales of vaccines by these stores in Nigeria, such as unstable power supplies, inadequate cold chain maintenance, and inefficient logistics systems, among other things.

The questionnaire captured data on sources of vaccines and vaccine cold chain. The study found that more than 84% of agro-veterinary stores sourced their vaccine through distributors or marketers, and only a small proportion sourced them from veterinary clinics (15.7%). This finding is consistent with another study that reported that there is limited access to, and availability of, retail outlets such as agro-veterinary stores with a new range of animal health products because they are only supplied by wholesalers ( Elelu, 2017). This is partly as a result of a decline in multi-national manufacturing companies that are relocating their business operations from most African countries, including Nigeria, and partly due to their focus on large-scale commercial companies as their market target ( Grasswitz *et al., *2004). The study showed that fewer than half (45.8%) of agro-veterinary outlets always store their vaccines in cold environments. This finding resonates with another study that reported that 61% of agro-veterinary stores could not maintain cold storage of their vaccines ( Chemuliti *et al.*, 2021). Maintenance of the cold chain during storage and logistics of the vaccine to livestock farmers was the main challenge reported (32.5%). This has been reported in previous studies as a challenge to the vaccine delivery system in low-income countries and the sale of effective vaccines in rural and peri-urban areas ( Adeiga and Harry, 2006; Ashok *et al., *2017; Chemuliti *et al., *2021). For the vaccine to be effective, pure, safe, and potent, cold chains must be maintained to confer protection against infection ( OIE, 2019). Lack of reliable power supply was reported as a constraint. Indeed, unreliable power supply is not unusual as more than a billion people around the world have no access to stable electricity and approximately 85% of them reside in rural areas of sub-Saharan Africa ( Babalola *et al.*, 2022). Other challenges listed have been previously reported by researchers who have studied agro-veterinary outlets in African countries, including Nigeria ( Grasswitz *et al.*, 2004; Elelu, 2017; Chemuliti *et al., *2021).

There were some limitations in the study. Some places known for security threats and insurgencies were purposely avoided within the states during this study. The sample size of 290 participants was smaller than initially intended.

### Recommendations

From participant suggestions ( Table 13) and the findings of the study, several recommendations can be made to improve vaccine distribution and usage:

1. Education of livestock farmers on the economic impacts of FMD and the cost-effectiveness of the use of FMD vaccine. The agro-veterinary store owners and workers should also be trained on the importance of cold chain for vaccine efficacy.
2. Deliberate and strategic awareness creation, product sensitization, and if possible, mass vaccination before embarking on sales. Partnership with the government is likely necessary for sensitization and awareness creation.
3. Provision of solar-powered refrigerators to agro-veterinary stores to ensure appropriate storage of the vaccine in an acceptable condition. Alternatively, the creation of mobile vaccination centers or veterinary clinics with refrigerators that can provide services to livestock farmers in rural and peri-urban areas.
4. Creation of sustainable vaccine distribution networks from the source (distributors or marketers) to the sink (livestock farmers), with smooth and rapid turn-around time and proven logistics that maintain cold chains. Demonstration to the smallholder farmers the possibility and benefits of an annual communal vaccination approach. Cheaper logistic arrangements should also be considered to reduce the cost of the vaccine for livestock farmers. Good road networks can also support this.
5. The vaccine must contain all the serotypes circulating in Nigeria to ensure effectiveness.
6. The price of the vaccine should be affordable. This can be achieved by partnership with the government livestock health programs.
7. Adoption of innovations such as thermo-stable FMD vaccine should also be considered if possible and available.

**Table 13.** Suggested improvements to vaccine distribution and usage.

Suggestion on improvement of vaccine distribution		
Suggestion	N=65	Explanation
**Solar powered refrigerator**	14	Provision of solar-powered refrigerator for vaccine storage/cold chain
**Provision of electricity**	8	Improvement in electric power supply, stable power supplier
**Provision of generator**	2	Provision of generator for storage
**Make vaccine available**	4	Available at the source, available to the agro-veterinary stores
**Education, sensitization, and awareness creation**	23	
**Transportation**	4	Cheap and affordable means of transportation, good road
**Cheap vaccine**	5	Government subsidy
**Accessibility**	11	Establish veterinary clinics/rural vaccine centers/mobile vaccine centers/clinic/storage facilities, support local market sellers, transport to the farmers, increase agro-veterinary stores
**Effective vaccine**	2	
**Thermostable vaccine**	2	Can be stored at room temperature
**Suggestion on improvement of vaccine usage by livestock farmers**		
**Suggestion**	N	Breakdown
**Education and sensitization**	23	Advert, mass vaccination, a collaboration of agro-veterinary with state vets, education of farmers
**Maintenance of cold chain**	6	Provision of facilities; education on the essence of the practice; monitoring of applications
**Availability of the vaccine at Agro-veterinary stores**	6	Consistent availability of the vaccine at agro-veterinary stores when demanded by the farmers; stores should ensure it is easily accessed by the farmers
**Price subsidy**	4	Government price subsidy; manufacturer’s consideration of price reduction
**Border control**	1	
**Effective vaccine**	1	

## Conclusion

To commence effective control of FMD in Nigeria, there is a need to improve the sales of vaccines by the agro-veterinary stores located in the rural and peri-urban areas. Many studies have considered the activities of agro-veterinary stores located in rural and peri-urban areas selling drugs, poultry and livestock vaccines, but this study, to the best of our knowledge, is the first report on their sales of FMD vaccine which is an important component of overall livestock disease control and prevention. There is a need to develop a supply chain that deeply entrenches agro-veterinary stores in the rural and peri-urban areas, to provide support in vaccine storage, in the form of solar-powered refrigerators or to establish mobile vaccination centers/veterinary clinics that service livestock farmers in rural and peri-urban areas, and ensure availability and accessibility of FMD vaccine to the farmers in these areas, who are the keepers of the most animal protein in Nigeria.

For a control program to be successful, there is the requirement to be intentional and to plan well-structured education programs, sensitization, and awareness creation campaigns on FMD and its control measures among livestock farmers through qualified agro-veterinary owners.

## Ethics and consent

There is no legal requirement in Nigeria for review by an Ethics Committee of such a study. However, since it was decided to aim to publish in Gates Open Research, it was felt that a retrospective review would be appropriate (rather than none at all). Ethical approval for the project, questionnaire, consent forms and other participant information materials was obtained from the University of Ibadan (UI) UCH Ethics Committee of the Institute for Advanced Medical Research and Training (IAMRAT), College of Medicine, University of Ibadan (registration number: NHREC/05/01/2008a; UI/UCH Ethics Committee assigned number: UI/EC/24/0256), dated 08/04/2024 to 07/04/2025.

Informed written consent was obtained from all participants before the questionnaire was administered.

## Data Availability

Harvard Dataverse: Foot-and-Mouth Disease (FMD) Vaccine Market Survey at Agro-Veterinary Stores in Rural and Peri-urban Areas of Nigeria. https://doi.org/10.7910/DVN/AJKGNS ( Sopeju, 2024). This project contains the following underlying data:
• Anonymised Agrovet Survey Data.xlsx Data are available under the terms of the Creative Commons Zero “No rights reserved” data waiver (CC0 1.0 Public domain dedication). Due to full data containing identifiable information, it has not been made publicly available. Researchers in a similar field can request further details of the interviews from the corresponding author, apeters@arpexas.com. Harvard Dataverse: Foot-and-Mouth Disease (FMD) Vaccine Market Survey at Agro-Veterinary Stores in Rural and Peri-urban Areas of Nigeria. https://doi.org/10.7910/DVN/AJKGNS ( Sopeju, 2024). This project contains the following extended data:
• Questionnaire.pdf Data are available under the terms of the Creative Commons Zero “No rights reserved” data waiver (CC0 1.0 Public domain dedication).
